# Parental leave during pediatric fellowship training: A national survey

**DOI:** 10.1371/journal.pone.0279447

**Published:** 2022-12-22

**Authors:** Nicolle F. Dyess, Blair W. Weikel, Jennifer M. Barker, Timothy P. Garrington, Thomas A. Parker

**Affiliations:** 1 Division of Neonatology, Department of Pediatrics, University of Colorado, Aurora, Colorado, United States of America; 2 Department of Pediatrics, University of Colorado, Aurora, Colorado, United States of America; 3 Division of Endocrinology, Department of Pediatrics, University of Colorado, Aurora, Colorado, United States of America; 4 Division of Hematology and Oncology, Department of Pediatrics, University of Colorado, Aurora, Colorado, United States of America; Boston Children’s Hospital, UNITED STATES

## Abstract

**Background:**

Until recently, no uniform requirements for parental leave (PL) existed in graduate medical education. We implemented a national survey, with the objective of ascertaining fellows’ perceptions of PL policies and their impact. This is the first study to focus exclusively on pediatric subspecialty fellows.

**Methods:**

An online survey instrument was created targeting pediatric fellows.

**Results:**

The survey was accessed by 1003 (25%) of the estimated 4078 pediatric subspecialty fellows and 853 (21%) submitted surveys. Respondent demographic data paralleled the data reported by the American Board of Pediatrics. Half of respondents did not know whether their program had a written PL policy. Over 40% reported ≥ 5 weeks of paid PL. Most indicated that fellows use vacation, sick leave, and unpaid time for PL. Almost half of respondents (45%) indicated that their program’s PL policy increases the stress of having a child. Fellows chose establishing/extending paid leave and intentionally fostering a more supportive program culture as the most crucial candidate improvements. The importance of equitable PL polices between parent fellows and co-fellows was an important theme of our qualitative data. Fellows feel there is a moral misalignment between the field of pediatrics’ dedication to maternal and child health and current PL policies governing pediatric trainees.

**Conclusions:**

PL policies vary widely among pediatric fellowship programs and are often not known by fellows. Fellows are not satisfied with PL policies, which often exacerbate stress for new parents and burden their co-fellows. Targeted modification of several aspects of PL policies may improve their acceptance.

## Introduction

For many years, postgraduate medical trainees and medical education leaders have sought to reform parental leave (PL) policies in graduate medical education (GME) [1–5]. With the recent attention to trainee well-being and burnout, interest in GME PL policies has grown, but has focused primarily on residents or has not differentiated fellows from residents [4, 6]. Among residents, longer PL is associated with increased breastfeeding rates [7–9] and improved maternal post-partum physical and mental health [9]. Additionally, in non-GME populations, longer PL is associated with decreased infant and child mortality [10–12], increased parental bonding [11], decreased intimate partner violence [10, 11], increased childhood vaccination rates [11], and decreased maternal and infant rehospitalization rates [11], with paid leave showing greater benefit than unpaid leave [13].

Until recently, the Accreditation Council for Graduate Medical Education (ACGME) stipulated only that programs inform trainee candidates of their leave policies and the impact of PL on the timing of program completion [14], allowing programs wide latitude in creating leave policies as long as they conform to subspecialty board accreditation requirements and state employment law. For example, the American Board of Obstetrics and Gynecology allows for 12 weeks leave during any year of training [15] whereas, previously, the American Board of Pediatrics (ABP) only allowed for 4 weeks before needing to extend the duration of training. Although a pediatric program director (PD) could submit a waiver on the trainee’s behalf asking for additional leave time, a survey of PDs found this was widely misunderstood and underutilized [16]. In addition, the ACGME and subspecialty boards have previously constrained how programs approach the duration of PL by setting strict criteria for months of training to qualify for program completion and specialty board certification [17].

In July 2020, The American Board of Medical Specialties (ABMS) announced a new, progressive PL policy, effective July 2021, that allows trainees a minimum of 6 weeks leave without extending training or exhausting vacation time [18]. Shortly thereafter, the ACGME instituted a revised medical, parental, and caregiver leave policy, effective July 2022, which follows the guidance set forth by the ABMS [19], allowing a minimum of six weeks paid leave at least once during an ACGME-accredited program with a minimum of an additional week of paid time off to be used outside the first six weeks during the same academic year.

The goal of our study was to ascertain fellows’ perceptions of the specific elements of PL policies in their pediatric fellowship programs and to understand the impact of those elements on fellows prior to the new ABMS and ACGME guidelines. We conducted a national survey of pediatric subspecialty fellows that addressed the specific hypotheses that PL policies not only vary widely among fellowship programs but are often not known by fellows and that fellows are not currently satisfied with PL policy at their institutions. The study serves as a baseline that can be used to describe how programs adapt to the new guidelines. Additionally, there are components of PL that will likely not change as a result of the new guidelines and will remain important areas of future advocacy.

## Materials and methods

We constructed a web-based survey in Research Electronic Data Capture (REDCap) [20, 21] hosted by the University of Colorado, following systematic approaches to survey design previously described in the literature [22–25] to optimize quality, reliability, and validity. The survey addresses the following research aims:

1. Describe the range of elements that constitute PL policy among pediatric fellowship programs and whether fellows are familiar with the PL policy at their program.
2. Determine fellows’ satisfaction with leave policy.
3. Identify elements of leave policy that, if implemented, might improve a trainee’s experience of having a child during training.
4. Understand the experience with parental leave during pediatric subspecialty training from the fellows’ perspective.

The survey instrument was pretested with informal interviews of medical education leaders, cognitive interviews of fellows, and retrospective interviews of junior faculty. We piloted the survey with the pediatric fellows at the University of Colorado (n = 87). Please reference the supplemental material for survey instrument and detailed survey creation, pretesting, and implementation procedures (S1–S3 Files).

For the national survey, we solicited the participation of pediatric fellows at any post-graduate level during the 2019–2020 academic year through direct, electronic contact with PDs and program coordinators (PCs) at all ACGME-accredited, non-military pediatric fellowship programs in the United States. Five survey solicitations were sent electronically over a 3-week period in May and June of 2020. Emails were sent either directly to fellows for whom programs had provided email addresses or to PDs and PCs to distribute to their fellows.

We compared the distribution of respondents’ demographics to the data reported by the ABP for pediatric fellows in 2019–2020 (n = 4078) [26] using chi-square tests and found no differences. As such, we did not weight responses from our survey. We used descriptive analyses for the multiple-choice questions regarding the construction of PL policies.

To determine fellows’ satisfaction with the PL policies at their program, we created a composite satisfaction score calculated as the mean of the responses to nine Likert-scale questions. Correlation testing among the nine questions yielded a Cronbach’s Alpha of 0.8 indicating good internal consistency [24]. A principal axis factor analysis with varimax rotation validated assigning a single PL policy satisfaction score. Differences in mean score between demographic characteristics and PL policy components were tested for significance using t-tests or ANOVA.

We presented fellows with seven potential improvements to pediatric fellowship programs that have been proposed previously in the literature [1–3]. An average priority score and standard deviation were calculated for each proposed change using a scale from 1 (not a priority) to 5 (essential priority). Fellows were also asked to rank their top 3 choices.

Lastly, open-ended responses describing a trainee’s experience with PL during fellowship were individually reviewed and coded by authors ND and TP via thematic analysis. The authors met sequentially to extract themes and create a coding dictionary (S4 File). Re-coding of the qualitative data using the coding dictionary resulted in an inter-coder reliability of 89%. Discrepancies were discussed and settled. Themes were reviewed and agreed upon by the remaining authors, and quotes illustrating each theme were chosen.

All statistical analysis was conducted in SAS 9.4 (Cary, North Carolina). Coding and thematic analysis were performed in Microsoft Excel. The research proposal and survey were exempted by the University of Colorado’s Institutional Review Board (#20–0088). The study’s purpose, voluntariness, and confidentiality were described in the welcome page of the survey (S1 File), and by completing entering the survey, respondents provided their written, informed consent to participate in this research study.

## Results

The survey was accessed by 1003 (25%) of the estimated 4078 pediatric sub-specialty fellows in the United States [26]. Of those that accessed the survey, 853/4078 (21%) submitted either fully or partially completed surveys. Many programs did not respond to our attempts to reach them, and we cannot estimate how many, if any, of those programs forwarded the survey to their fellows. As we do not know the number of fellows who received the survey, we cannot calculate a true response rate. Estimates of the response rate are further discussed in S3 File.

**Fig 1:**
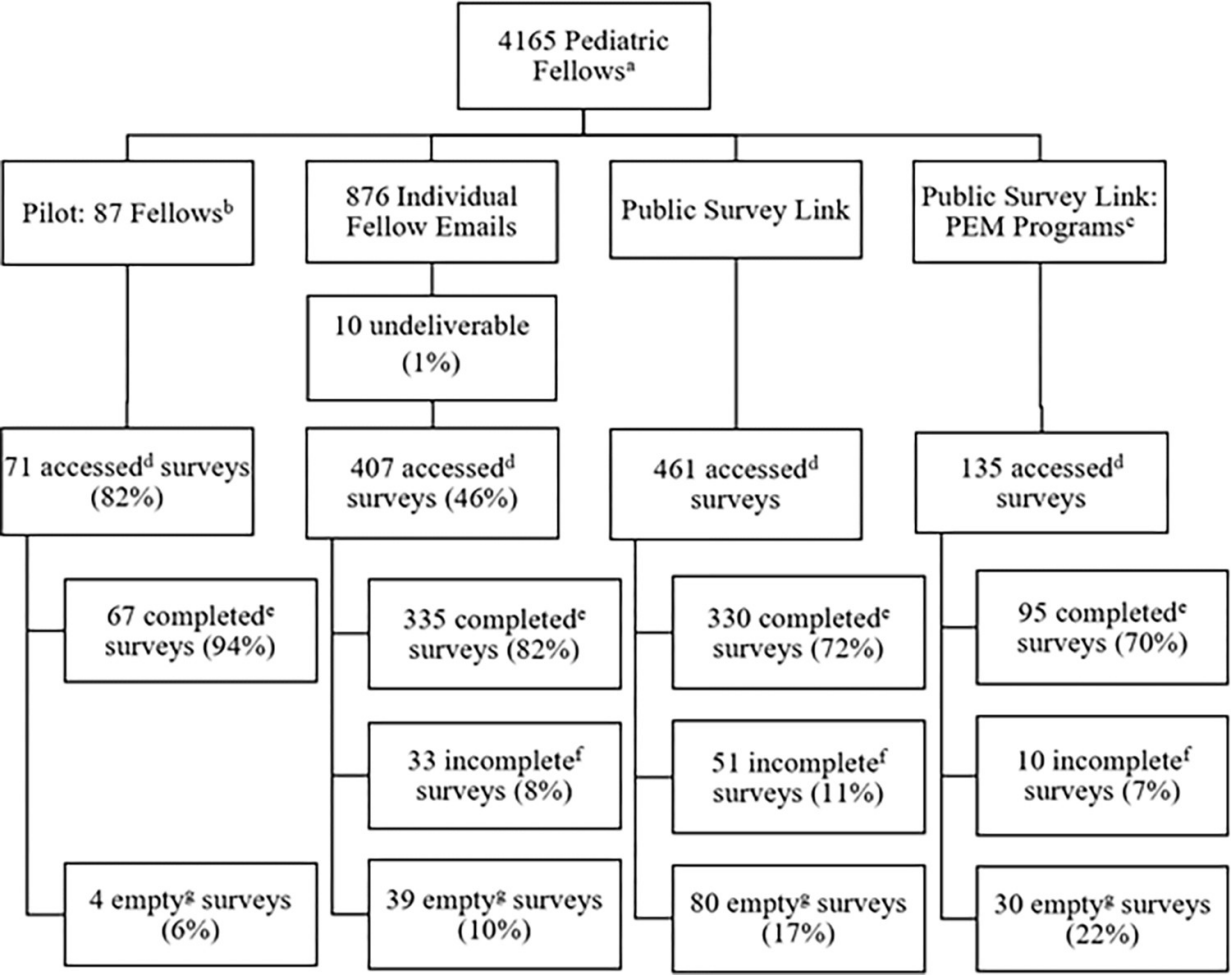
Breakdown of survey responses. Abbreviations: Pediatric Emergency Medicine (PEM). ^a^ American Board of Pediatrics 2019–2020 fellowship data. ^b^ Pediatric fellows at the University of Colorado. Data from the pilot study is not included in the data analysis of this manuscript. ^c^ The survey was distributed separately to PEM fellows via the PEM program director listserv. ^d^ An accessed survey is one where a respondent has clicked on the survey link and proceeded to the survey questions, after reviewing the welcome page of the survey. ^e^ A completed survey is one that was accessed, answered, and submitted. ^f^ An incomplete survey is one that was accessed and partially completed but not submitted. Partial answers were included in the data analysis of this manuscript. ^g^ An empty survey is one that was accessed but left unanswered for all questions.

The response breakdown is depicted in Fig 1, and the characteristics of the fellows who completed the survey are listed in Table 1.

**Table 1:** Characteristics of respondent fellow sample^a^.

	National Survey Data^b^	ABP 2019 Fellowship Data [26] (N = 4165)
Characteristic (total responses)	N (%)	N (%)
Subspecialty (N = 747)		
Adolescent Medicine	18 (2)	91 (2)
Cardiology	45 (6)	458 (11)
Child Abuse	9 (1)	45 (1)
Critical Care	91 (12)	552 (13)
Developmental Pediatrics	33 (4)	120 (3)
Emergency Medicine	100 (13)	558 (13)
Endocrinology	57 (8)	227 (5)
Gastroenterology	56 (7)	325 (8)
Hematology/Oncology	53 (7)	482 (12)
Infectious Disease	49 (7)	167 (4)
Neonatology	148 (20)	777 (19)
Nephrology	31 (4)	109 (3)
Pulmonology	32 (4)	168 (4)
Rheumatology	25 (3)	86 (2)
Region (N = 699)		
Northeast	140 (20)	1079 (26)
Midwest	220 (31)	998 (24)
South	227 (32)	1353 (32)
West	112 (16)	735 (18)
Program Size (N = 744)		
1–6 Fellows	390 (52)	1938 (47)^c^
≥ 7 Fellows	354 (48)	2228 (53)^c^
Gender (N = 757)		
Female	576 (76)	2834 (68)
Male	176 (23)	1331 (32)
Other^d^	6 (1)	--
Educational Debt (N = 744)^d^		
$0	212 (28)	--
≤$200,000	143 (19)	--
$200,001 - $300,000	221 (30)	--
>$300,000	168 (23)	--
Have Children (N = 751)^d^		
Yes	325 (43)	--
No	426 (57)	--
Had child during fellowship (N = 324)^d^		
Yes	173 (53)	--
No	151 (47)	--

Abbreviations: American Board of Pediatrics (ABP).

^a^ National survey data does not include the 67 pediatric fellows at the University of Colorado who completed the pilot study.

^b^ Excludes data from the pilot study of pediatric fellows at the University of Colorado (n = 67).

^c^ ABP program size numbers do not add up to the total number of pediatric fellows (N = 4165) as there was a trainee that switched programs in the middle of academic year and as such, fits under two different program size categories.

^d^ Data is not collected by the ABP.

### Research aim 1: Fellows’ understanding of parental leave elements

Approximately half (48%, 441/853) of respondents did not know whether their fellowship program had a written policy governing PL. Of the respondents who reported on how well the PL policy of their program met the needs of its trainees (from 1 indicating “never” to 5 indicating “always”), the median response was a 3 (IQR [2, 3]). Only 45% (196/434) stated their needs were being met “often” or “always.”

Half of respondents (433/851) did not know how many weeks of paid PL their program allowed. The distribution of paid weeks allowed by program, as indicated by the half who did endorse knowing the policy, is depicted in Table 2 along with information on whether vacation/holiday time, sick time, elective time, and/or unpaid leave is utilized.

**Table 2:** Elements of parental leave policy.

	Total
	N (%)
How many weeks of paid leave does your program allow for parental leave? (excluding vacation/holiday, sick days, or elective time) (N = 851)	
I don’t know	433 (51%)
Other response	418 (49%)
0 weeks	108 (26%)
1–2 weeks	52 (12%)
3–4 weeks	83 (19%)
5–6 weeks	111 (27%)
7–8 weeks	40 (10%)
≥9 weeks	24 (6%)
Do fellows at your program apply their available vacation/holiday time to construct or extend their parental leave? (N = 848)	
I don’t know	223 (26%)
Other response	625 (74%)
Yes	598 (96%)
No	27 (4%)
Do fellows at your program apply their available sick time to construct or extend their parental leave? (N = 744)	
I don’t know	277 (37%)
Other response	467 (63%)
Yes	263 (56%)
No	44 (9%)
We don’t get sick days	160 (34%)
Do fellows at your program sometimes take unpaid leave to construct or extend their parental leave? (N = 844)	
I don’t know	481 (57%)
Other response	363 (43%)
Yes	264 (73%)
No	99 (27%)
Does you fellowship program utilize a designated scholarly/elective rotation with limited productivity expectations to construct or extend parental leave? (N = 843)	
I don’t know	343 (41%)
Other response	500 (59%)
Yes	288 (58%)
No	212 (42%)
Are fellows who take parental leave expected to make up any missed clinical responsibilities? (N = 847)	
I don’t know	338 (40%)
Other response	509 (60%)
Yes	389 (76%)
No	120 (24%)
Do fellows often take on a greater than usual load of clinical responsibilities during or after pregnancy to “pay for” their parental leave? (N = 845)	
I don’t know	343 (41%)
Other response	502 (59%)
Yes	289 (58%)
No	213 (42%)

### Research aim 2: Fellows’ satisfaction with parental leave policy

Eighty-six respondents’ answers were excluded as they indicated “no opinion” for all questions in this section, leaving responses from 626/853 (73%) of all respondents to create satisfaction scores. The mean satisfaction score of the cohort was 3.0 (SD = 0.7), with 1 indicating “very dissatisfied” and 5 indicating “very satisfied”, and did not differ by program subspecialty, region, or size; level of fellow’s educational debt; or whether the fellow had children (S5 File).

Almost half of respondents (45%, 283/626) indicated that the PL policy at their program increases the stress of having a child during training. Additionally, a third of respondents (206/626) stated the PL policy at their program negatively impacts the experience of having a child during training. Fellows with children were more likely to respond to this portion of the survey than fellows without children (292/325, 90% vs 298/426, 70%, p < 0.0001).

Satisfaction scores correlated with many aspects of how fellows reported PL is constructed at individual programs. Higher satisfaction scores correlated with a PL policy that was written and available, one that explicitly applies equally to fathers/partners and adoption/fostering, one constructed from more weeks of dedicated paid PL rather than use of other forms of time off (vacation/holiday/sick/unpaid time), one that allows the use of an elective to extend PL, one that forgives missed clinical time, and one that does not require increased clinical workloads before/after to “pay for” PL (S5 File). Those who felt that the PL policy at their institution was meeting the needs of its trainees had higher satisfaction scores.

### Research aim 3: Proposed changes to parental leave policy

**Fig 2:**
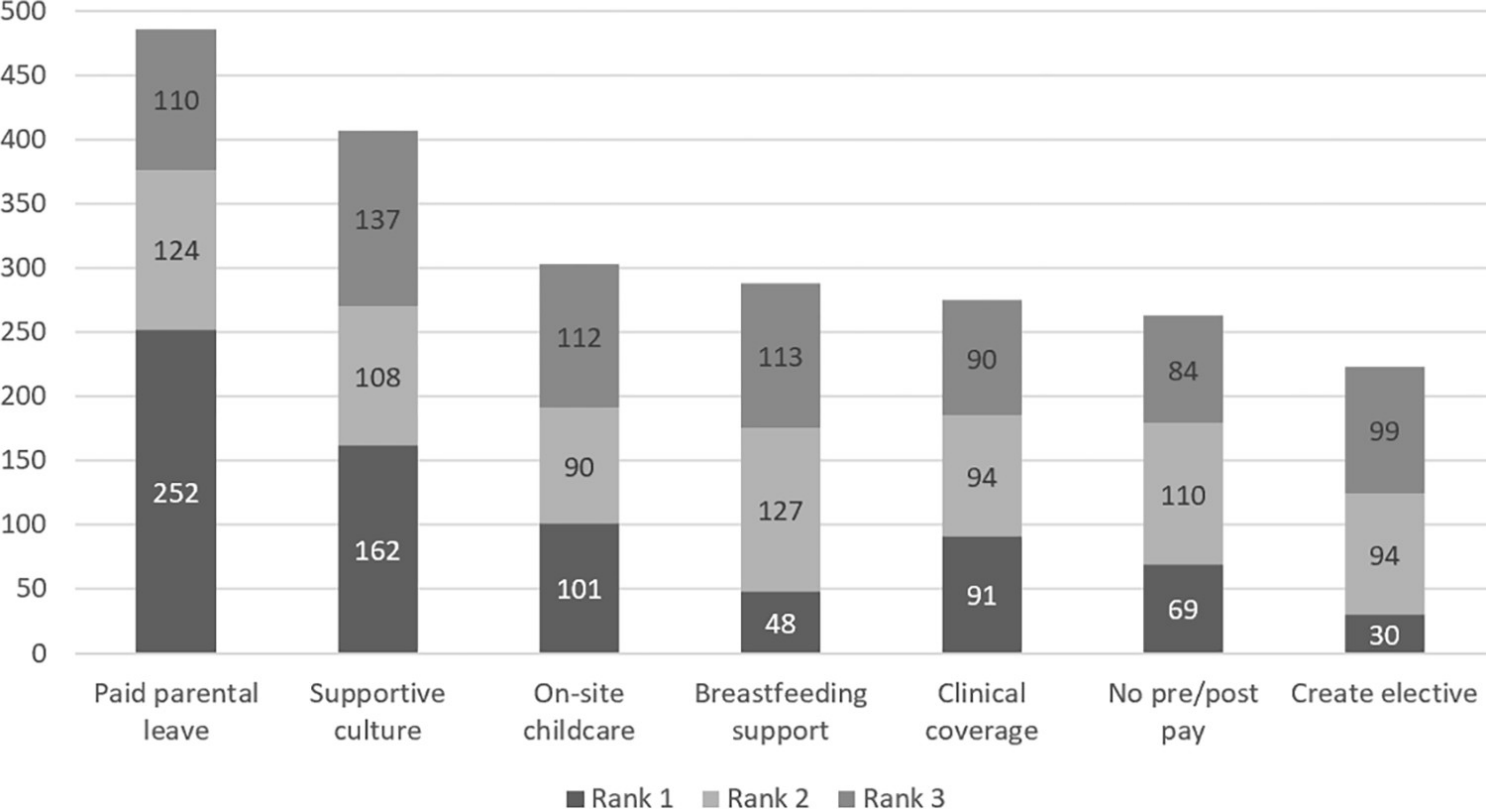
Ranking of the proposed changes to fellowship programs.

Mean priority scores for the 7 proposed changes are shown in Table 3. Ranking results of the proposed changes are shown in Fig 2. Establishing/extending paid PL and intentionally cultivating a program culture that supports PL were ranked the highest.

**Table 3:** Proposed changes to pediatric fellowship programs and their average priority score.

Proposed Change (N)	Average Priority Score^a^ (SD)
Fellowship programs should improve workplace support for breastfeeding/pumping breast milk. (740)	4.5 (0.7)
Fellowship programs should intentionally cultivate a culture among faculty and fellows that supports parental leave. (748)	4.4 (0.7)
Fellowship programs should establish/extend paid parental leave. (723)	4.3 (0.8)
Fellowship programs should establish on-site childcare facilities. (737)	4.0 (0.9)
Fellowship programs should assume responsibility for arranging clinical coverage for the fellow during parental leave. (742)	3.9 (0.9)
Fellowship programs should establish a formal elective (ie, homecare elective) for new parents with minimal scholarly expectations. (725)	3.7 (1.1)
Fellowship programs should relieve the fellow of the responsibility to prepay or repay missed clinical time during parental leave. (737)	3.5 (1.2)

^a^ Priority scores were calculated using a scale from 1 (not a priority) to 5 (essential priority).

### Research aim 4: Trainee’s experience with parental leave during fellowship

Many respondents were parents (43%, 325/751) and reported that daycare (50%, 161/325) and a fellow’s partner (38%, 123/325) were the primary means of childcare. A quarter of all respondents (26%, 196/751) planned on having a/another child during fellowship, and 16% (125/751) were unsure of their parental plans to have children. Half of respondents felt that fellowship (26%, 197/753) or post-fellowship (25%, 195/753) was the best time to have children. Few (10%, 73/749) considered PL policy when choosing fellowship programs.

Of those who were not planning on having a child during fellowship (57%, 430/751), 17% (71/430) stated their program’s PL policy was a deterrent. A substantial number of respondents without children who experienced a colleague have a child during fellowship (25%, 57/237) felt unduly burdened by a colleague’s decision to have a child during fellowship. Fewer (13%, 30/237) felt their colleagues should have delayed childbearing.

Of respondents who had children during fellowship (50%, 173/325), the majority breastfed their children (81%, 140/173). Most did not experience significant financial difficulties (83%, 140/169). Over a third (36%, 63/173) reported not receiving adequate PL policy information prior to their leave, emphasizing a lack of clarity, accessibility, and consistency. One fellow stated that their “biggest stressor was minimal communication from [their] program” and another reported that their program “[applied] different rules of an unclear source to different fellows.” Sadly, 15% (26/170) of respondents reported discrimination at work while pregnant. One fellow reported that they were “told by [their] program that if [they] had a second pregnancy…[that they] would not be allowed to graduate” causing that fellow to “conceal [their] pregnancy until almost [their] third trimester for fear of this.” Approximately a third took unpaid leave (4 weeks on average) to construct/extend their PL (28%, 44/158), and 35% (60/172) are having to extend fellowship due to their leaves. Regardless, 87% (150/172) do not wish they had delayed childbearing until after fellowship.

Seven themes emerged from the qualitative data. The themes and representative quotes can be seen in Table 4. Many fellows remarked on the impact of laws and policies emphasizing “[America] is decades behind the rest of the world” and that “[ACGME] rules truly deter trainees from having children during their training.” Equity, especially between fellows, and burden, both on co-fellows and parent fellows, were also important themes. One fellow suspected that their program’s approach to parental leave was a “contributing [factor] to [their fetal] demise” while another fellow proclaimed that “meanwhile, [their] program is making great concessions for someone to spend 5.5 months in Africa…[with] reduced call responsibilities.” Several pediatric fellows felt “morally responsible for leading the way on improving family leave” and current training policies left them feeling “embarrassment [for being] a pediatrician.”

**Table 4:** Themes from qualitative analysis and representative quotes.

THEME	REPRESENTATIVE QUOTES		
**Impact of Laws and Culture**	“How America deals with maternity leave is a crime.”	“The only thing I am concerned about are families that have children during the first year of fellowship because FMLA does not start until working a job for 12 months at our institution.”	“We are decades behind the rest of the world in our respect and care for working mothers!!”
**Impact of Policies Governing Pediatric Trainees**	“That is where the real problem lies in the ACGME policies. Their rules truly deter trainees from having children during their training.”	“The AAP needs to stop requiring mandatory make up for parental leave.”	“Many associations (AAP, ABEM, etc) have their own, and different, policies on parental leave which may even differ from the institutional policy.”
**Equity**	“Meanwhile, my program is making great concessions for someone to spend 5.5 months in Africa during a fellow’s second year as part of a research project. This fellow is having reduced call responsibilities while I took all the backup I was suppose to and all of the calls.”	“It’s absurd to expect other fellows to perform additional duties in order to support one fellow’s CHOICE to have children during fellowship. . .Where the hell is the equity in that?”	“I also feel that fellows should not be excused from all call burden or responsibility because of leave. I feel it is my responsibility as a member of the program and I owe it to my own training to "pay back" at least some calls at some point. It may not be exactly equal, but so long as it is fair, I am fine doing so.”
**Burden on Co-Fellows**	“When my co-fellow took maternity leave, I had night call almost every single night with no help from attendings for weeks.”	“In programs with small number of fellows- there is the added guilt of having children because all of the calls will have to be taken by your co-fellows. This is a major factor for me when it comes to planning a pregnancy.”	“So fellows have to work extra hours to cover for their colleague for weeks at a time. This is absurd, unfair. . . . and the norm.”
**Burden on Parent Fellows and Their Families**	“We are single income with 3 kids. That was not doable, so I took my 3 sick days and went back to work. While I’ve loved my fellowship, I will never find that morally acceptable that I was forced to do that.”	“Stress about parental leave in fellowship is a huge factor in my current decision to not have a child in training…”	“I suspect that the stress of fellowship in a malignant program, having to front-load my schedule in preparation for "maternity leave", and a heavier than normal work-load due to fellow shortage and the program’s dependence on fellows were contributing factors to the [fetal] demise.”
**Burden on Pediatric Medical Professionals**	“I think academic medical centers and training programs are morally responsible for leading the way on improving family leave. We’re a Children’s hospital!!! What does 2 weeks of paid leave say about how our institution cares for children and families?”	“It is an embarrassment to be a pediatrician, attempting to establish breast-feeding, and still only having six weeks to do so. And I am having to use all of my vacation time. . .If a parent asked me my professional opinion. . .I would tell them to get a new job!”	“As a pediatrician, parenthood truly is one of the most educational experiences of my life and has made me a much better clinician. Possibly more than any other 4 weeks of my training.”
**Programmatic Approaches to Parental Leave**	“There is a difference between policy of my department, graduate medical education, and the hospital. This was a challenging process to navigate. . .I am clearly not the first fellow to have a baby, so I am not sure why it is not more straightforward.”	“No one can legally stop me from taking 12 weeks leave, but I have been told multiple times that MOST trainees do not take this much time but it’s fine IF MY DOCTOR DEEMS IT MEDICALLY NECESSARY. It is treated as if I’m taking a prolonged vacation.”	“Our program does a lot of under the table stuff to help us extend our "leave" to up to 12 weeks. . .My program director tries really hard to mitigate that with fake research electives and not double checking if we take an extra week of vacation and things like that, but none of that shady business would be necessary if [our program] established proper parental leave and childcare options.”

## Discussion

We found that many pediatric trainees have children or are planning to have a child during fellowship. Fellows report wide variability in how PL is constructed, and nearly half of fellows are uncertain whether their program has a formal PL policy. Many fellows are not satisfied with their program’s current PL policies, which they view as fostering stress, unduly burdening co-fellows, and negatively impacting the experience of having a child. Fellows prioritize establishing/extending paid PL and intentionally fostering a culture supportive of PL among faculty and fellows as the top candidates for improvement. The importance of equitable PL policies, mindful of both parent fellow and co-fellow burden, was an important theme of our qualitative data. Fellows expressed distress over the moral discrepancy between the field of pediatrics’ dedication to maternal and child health and current PL policies governing pediatric trainees.

This cross-sectional survey study yields important and new information because of its focus on subspecialty trainees. Most of the literature on PL policy in GME has focused on residents or has not distinguished between residents and fellows. Although PL presents residents and fellows with many shared challenges, including the financial hardships of taking unpaid leave and difficulty with securing childcare, fellowship is unique in that a substantial portion of time is spent pursuing scholarly work. Time spent in scholarly work may be more flexible for scheduling parental leave. However, fellowship occurs later in one’s training, potentially increasing the urgency for family planning for many trainees given the age limits of fertility. Fellows who aspire to academic careers also face pressure to demonstrate scholarly productivity to improve competitiveness for academic positions. Lastly, any burden created by PL for other trainees is likely greater compared to residency programs because there are typically fewer fellows to share that burden. This last point was articulated as a barrier to PL by fellowship PDs in a recent study [27].

Over 40% of our respondents reported ≥ 5 weeks of paid PL, considerably more than the average of 3 weeks reported for pediatric residencies in 2007 [28]. Our findings align with more recent studies of PL policy during residency [4, 7, 9]; however, those studies do not indicate whether vacation, holiday, sick, and/or elective days are allocated to “paid PL.” Our survey sought to isolate dedicated, paid time off for PL from all other categories of paid time off. Despite reporting considerable paid PL, respondents indicated that most fellows use vacation, holiday, sick, and unpaid days for PL which is a similar finding in a recent study of a subset of internal medicine and pediatric fellowship programs by Jamorabo et al. [27]. Additionally, most fellows report they are expected to make up time and take on greater workloads before/after the arrival of a child to “pay for” their PL. Having to consolidate clinical responsibilities may create an additional burden at a time of particular vulnerability, immediately before and after the arrival of a child.

Previous studies have sought to determine how well residents understand their institution’s existing PL policy [7, 29, 30]. In a 2006 national survey, 90% of pediatric residency PDs reported the existence of a formal PL policy at their institutions [28], and 88% of graduating pediatric residents reported being aware of these policies [31]. In contrast, nearly 50% of our respondents reported they were not aware of a formal PL policy. In the study by Jamorabo et al. [27], 72% of fellows stated their fellowship program had a formal leave policy, and the majority did not feel their institution’s policies were easily accessible, even upon request. Programs have the opportunity to improve communication and access to information about PL policies and to convey a recognition of the import and value of PL. A readily accessible policy might allow fellows and program leaders to have more constructive discussions when planning for leave, fewer uncomfortable requests for information when the trainee may not yet be ready to disclose their family planning, improved planning on behalf of the trainee, and increased awareness among all fellows, including colleagues not going on leave, of what to expect and prepare for during a co-fellow’s leave.

In our study, pediatric fellows report a lack of satisfaction with current PL policy. Despite the increased schedule flexibility in fellowship, concern about the adequacy of PL during fellowship might lead to deferred childbearing until after training when PL may be more substantial [32]. Deferring childbearing increases stress due to fears of increased infertility and obstetrical complication rates associated with advancing maternal age [33, 34].

We asked respondents to consider a number of potential changes that might alleviate the stress of having or adopting a child during training. Among these, the most popular was establishing/extending paid PL, a change that has now taken place following the new ABMS and ACGME guidelines [18, 19]. Our respondents also gave high priority to several other more modest and considerably less expensive changes, including fostering a program culture more supportive of PL, program assumption of responsibility for arranging for clinical coverage during PL, and improved breastfeeding support. Similarly, in the study by Jamorabo et al. [27], one fifth remarked that the culture of medicine is the biggest barrier to PL support. Our respondents described their programs as “actively discourage[ing]” and “[having] so many negative attitudes towards [PL].” Strategies to augment a culture of transparency and support including having a clear, accessible PL policy to decrease the stigma which surrounds childbearing in training. Stigma surrounding childbearing in training and discrimination experienced by physician and trainee parents have been described previously including lower peer evaluation scores in the postpartum period [35], disrespectful comments from faculty and peers regarding the time taken for maternity leave and/or breastmilk pumping [36, 37], and fear that childbearing will adversely affect their colleagues’ professional perceptions of them and their future careers [33, 34, 36, 38, 39]. Increased schedule flexibility [34], attention to improving self-care [40], and efforts to mitigate burnout [41] have also been shown to promote a supportive culture [42]. In addition, the perception of a supportive culture is further enhanced by encouraging longer leaves, regardless of pay, and providing personal mentorship [6, 27, 42].

Our analysis of the survey’s open-ended questions revealed several themes. Many comments expressed grievance with the lack of broad consensus in support of routine parental leave in the United States. Fellows particularly faulted those policy-making bodies governing pediatric medical education (ACGME, ABP, ABMS, local GME offices) for creating confusion and inconsistencies. Many fellows objected to the lack of a well-defined PL policy in their own program, sometimes leading to creation of PL that felt ad-hoc and capricious. The burden of shifting missed work during leave to co-fellows created feelings of guilt for parent fellows. Many of those co-fellows voiced feeling resentment for having to assume additional work and suggested that faculty should share some responsibility; others stated that PL time that resulted in fewer required months of training created inequities between those that took PL and those that didn’t. This theme of increased co-fellow burden is particularly interesting in light of a recent study which found that a majority of fellowship PDs felt that there was no increased burden upon co-fellows [27]. Lastly, fellows frequently alluded to a special responsibility for training programs in pediatrics to be national leaders in supporting robust leave policies for their trainees given the demonstrated positive impact that extended paid leave has on the health of children and their parents.

In addition to possible sources of survey error addressed in S3 File, there were other limitations to our study. As the construction of PL varies widely among programs, it is likely we did not ask about all possible variations and strategies implemented by some programs (ie, short- and long-term disability insurance, use of locum physicians to assist with clinical coverage, etc.). We also did not include questions concerning health insurance policies and how they may affect trainee satisfaction with PL policy. Additionally, fellows were clustered by fellowship program and institution, and we were unable to account for this clustering in our analysis of the data. We provide reliability and validity evidence in S2 and S3 Files.

For future directions of this study, it would be worthwhile to repeat the survey in several years to determine the effects of the changes to PL policy in response to the new ABMS and ACGME guidelines. Additionally, a future survey of fellowship PDs would describe their new baseline practices and perceived barriers to change.

## Conclusion

PL policies vary widely among pediatric fellowship programs and are often not known by fellows. Our results indicate fellows were not satisfied with PL policies in fellowship prior to recent mandates and that there are several areas that would improve the experience of PL for fellows.

## Supporting information

S1 File The REDCaP survey instrument. (DOCX)

S2 File Pretesting procedures. (DOCX)

S3 File Implementation and estimating response rate. (DOCX)

S4 File Coding dictionary. (DOCX)

S5 File Comparing mean satisfaction scores to components of parental leave and to fellow characteristics. (DOCX)

## Abbreviations

ABMS: American Board of Medical Specialties
ABP: American Board of Pediatrics
ACGME: Accreditation Council for Graduate Medical Education
GME: Graduate Medical Education
PC: Program Coordinator
PD: Program Director
PEM: Pediatric Emergency Medicine
PL: Parental Leave
REDCap: Research Electronic Data Capture
