# Supplementary material for: Parental leave during pediatric fellowship training: A national survey
Source: PLoS One. 2022 Dec 22;17(12):e0279447. doi: 10.1371/journal.pone.0279447 (PMC9779013; doi:10.1371/journal.pone.0279447)
Supplement: S1 File — (DOCX) [file pone.0279447.s001.docx]

**Supplement 1: The REDCaP Survey Instrument**

[PAGE ONE]

**The Effects of Parental Leave Policies in Pediatric**

**Fellowships on Trainees**

Welcome to The Effects of Parental Leave Policies in Pediatric Fellowships on Trainees Survey!

You are being asked to be in this research study because you are a current pediatric fellow.

If you join the study, you will be asked to complete this brief survey which is being conducted to understand how

pediatric fellows feel about the parental leave policies at their programs. We are interested in the views of all

trainees regardless of whether they have OR do not have children.

Possible discomforts or risks include recalling negative past experiences. There may be risks the researchers have not thought of.

This study is not designed to benefit you directly.

Every effort will be made to protect your privacy and confidentiality. No personally identifiable information will be

associated with your responses in any reports of the data. We will delete your email upon completion of the survey.

You have a choice about being in this study. You do not have to be in this study if you do not want to be.

The data we collect will be used for this study but may also be important for future research. Your data may be used for future research or distributed to other researchers for future study without additional consent if information that identifies you is removed from the data.

If you have questions, you can e-mail or call Nicolle Dyess, the study director, at nicolle.dyess@childrenscolorado.org or nicolle.dyess@cuanschutz.edu. You can contact her to ask questions at any time.

You may have questions about your rights as someone in this study. If you have questions, you can call COMIRB (the responsible Institutional Review Board) at (303) 724-1055.

By completing this survey, you are agreeing to participate in this research study.

Thank you,

Nicolle Dyess, MD

Study Director

Neonatology Fellow

University of Colorado

Tom Parker, MD

Project Mentor

Neonatology Program Director

University of Colorado

[PAGE TWO]

How often is the parental leave policy at your program meeting the needs of its trainees?

- Always
- Often
- Sometimes
- Rarely
- Never
- I do not know

Does your fellowship program have a written policy governing parental leave?

- Yes
- No
- I do not know.

Does the parental leave policy at your program apply equally to mothers and fathers/partners?

- Yes
- No
- I do not know

Does the parental leave policy at your program apply equally to childbearing and adoption/fostering?

- Yes
- No
- I don't know

How many weeks of paid leave does your program allow for parental leave? Do not include vacation/holiday, sick

days, or elective time.

- 0 weeks
- 1-2 weeks
- 3-4 weeks
- 5-6 weeks
- 7-8 weeks
- > 9 weeks
- I do not know

Do fellows at your program apply their available vacation/holiday time to construct or extend their parental leave?

- Yes
- No
- I don't know

Do fellows at your program apply their available sick time to construct or extend their parental leave?

- Yes
- No
- We do not get sick days
- I don't know

Do fellows at your program sometimes take unpaid leave to construct or extend their parental leave?

- Yes
- No
- I don't know

Does your fellowship program utilize a designated scholarly/elective rotation with limited productivity expectations to construct or extend parental leaves?

- Yes
- No
- I don't know

Are fellows who take parental leave expected to make up any missed clinical responsibilities?

- Yes
- No
- I don't know

[BRANCHING LOGIC: IF YES, DISPLAY THIS QUESTION]

Which of the following clinical responsibilities need to be made up? Check all that apply.

- In-house call shifts
- At-home call shifts
- Inpatient service time
- Outpatient clinic time
- Elective time
- Research time

Do fellows often take on a greater than usual load of clinical responsibilities during or after pregnancy to "pay for"

their parental leave?

- Yes
- No
- I don't know

Who is responsible for identifying colleagues to cover missed calls, clinics, or service times during a fellow's parental leave?

- The fellow
- The program
- Both
- I don't know

[PAGE THREE]

How fair or unfair is your program's parental leave policy?

- Very fair
- Fair
- Neither fair nor unfair
- Unfair
- Very unfair
- No opinion

How easy to understand or difficult to understand is your program's parental leave policy?

- Very easy to understand
- Easy to understand
- Neither easy to understand nor difficult to understand
- Difficult to understand
- Very difficult to understand
- No opinion

How positively or negatively does your program's parental leave policy impact a fellow's experience with having a

child?

- Very positively impacts
- Positively impacts
- Neither positively impacts nor negatively impacts
- Negatively impacts
- Very negatively impacts
- No opinion

To what extent does your program's parental leave policy increase or decrease the stress of having a child during

training?

- Significantly increases the stress
- Increases the stress
- Neither increases nor decreases the stress
- Decreases the stress
- Significantly decreases the stress
- No opinion

How positively or negatively does your program's parental leave policy impact a fellow's career advancement?

- Very positively impacts
- Positively impacts
- Neither positively nor negatively impacts
- Negatively impacts
- Very negatively impacts
- No opinion

How sufficient or insufficient is your program's parental leave policy for the establishment of breastfeeding?

- Very sufficient
- Sufficient
- Neither sufficient nor insufficient
- Insufficient
- Very insufficient
- No opinion

To what extent does your program's parental leave policy encourage or discourage trainees from having a child

during fellowship?

- Strongly encourages
- Encourages
- Neither encourages nor discourages
- Discourages
- Strongly discourages
- No opinion

How sufficient or insufficient is your program's parental leave policy for the establishment of adequate, post-partum self-care?

- Very sufficient
- Sufficient
- Neither sufficient nor insufficient
- Insufficient
- Very insufficient
- No opinion

How positively or negatively does your program's parental leave policy impact your ability to provide excellent

patient care?

- Very positively impacts
- Positively impacts
- Neither positively nor negatively impacts
- Negatively impacts
- Very negatively impacts
- No opinion

[PAGE FOUR]

The following are potential changes to pediatric fellowship programs that have been proposed to minimize the stress of having or adopting a child during training. To what extent do you feel the proposed changes are or are not a priority:

Fellowship programs should assume responsibility for arranging clinical coverage for the fellow during parental leave.

- Not a priority
- Low priority
- Neutral
- High priority
- Essential priority
- No opinion

Fellowship programs should relieve the fellow of the responsibility to prepay or repay missed clinical time during

parental leave.

- Not a priority
- Low priority
- Neutral
- High priority
- Essential priority
- No opinion

Fellowship programs should intentionally cultivate a culture among faculty and fellows that supports parental leave.

- Not a priority
- Low priority
- Neutral
- High priority
- Essential priority
- No opinion

Fellowship programs should improve workplace support for breastfeeding/pumping breast milk.

- Not a priority
- Low priority
- Neutral
- High priority
- Essential priority
- No opinion

Fellowship programs should establish a formal elective (ie, "homecare elective") for new parents with minimal

scholarly expectations.

- Not a priority
- Low priority
- Neutral
- High priority
- Essential priority
- No opinion

Fellowship programs should establish/extend paid parental leave.

- Not a priority
- Low priority
- Neutral
- High priority
- Essential priority
- No opinion

Fellowship programs should establish on-site childcare facilities.

- Not a priority
- Low priority
- Neutral
- High priority
- Essential priority
- No opinion

**Of the stated potential improvements, rank your top 3.**

**Click "First Choice" next to your top choice, a "Second Choice" next to your second choice, and**

**a "Third Choice" next to your third choice. Leave the remaining blank.**

1. First Choice 2. Second Choice 3. Third Choice

Assume responsibility for

arranging clinical coverage for

the fellow during parental leave.

Relieve the fellow of

responsibility to prepay or repay

missed call during parental

leave.

Intentionally cultivate a culture

among faculty and fellows that

supports parental leave.

Improve workplace support for

breastfeeding.

Establish a formal elective (ie,

"homecare elective") for new

parents with minimal scholarly

expectations.

Establish/extend paid parental

leave.

Establish on-site childcare

facilities.

Is there another improvement that is not listed above that you believe would improve parental leave during

fellowship?

[FREE TEXT]

[PAGE FIVE]

Lastly, we would like to ask a few personal questions to help us further describe the impact parental leave policies

during fellowship have on fellows. We really appreciate your help and that of thousands of others who have been

asked to complete this national survey.

How old are you?

__________________________________

(years)

What is your gender identification?

- Male
- Female
- Transgender male
- Transgender female
- Gender variant/non-conforming
- Other
- Prefer not to answer

What is your ethnicity? You may choose more than one response.

- White
- Hispanic/Latino(a)
- Black/African American
- Native American/American Indian
- Asian/Pacific Islander
- Other
- Prefer not to answer

What is your fellowship specialty?

- Adolescent Medicine
- Cardiology
- Child Abuse
- Critical Care
- Developmental Pediatrics
- Emergency Medicine
- Endocrinology
- Gastroenterology
- Hematology/Oncology
- Infectious Disease
- Nephrology
- Neonatology
- Pulmonology
- Rheumatology

What region is your program in?

- New England (CT, MA, ME, NH, RI, VT)
- Mid Atlantic (NJ, NY, PA)
- East North Central (IL, IN, MI, OH, WI)
- West North Central (IA, KS, MN, MO, ND, NE, SD)
- South Atlantic (DC, DE, FL, GA, MD, NC, SC, VA, WV)
- East South Central (AL, KY, MS, TN)
- West South Central (AR, LA, OK, TX)
- Mountain (AZ, CO, ID, MT, NM, NV, UT, WY)
- Pacific (AK, CA, I, OR, WA)
- Territory (PR)

What year are you currently in your fellowship program?

- 1st year
- 2nd year
- 3rd year
- 4th year or greater

What is the total number of current fellows in your program?

__________________________________

(fellows)

Do you have any children?

- Yes
- No

[BRANCHING LOGIC: IF NO, DISPLAY THE FOLLOWING QUESTIONS 🡪]

Have any of your co-fellows had or adopted a child during fellowship?

- Yes
- No

[BRANCHING LOGIC: IF YES, DISPLAY THE FOLLOWING QUESTIONS 🡪]

Did you ever feel unduly burdened by your co-fellow(s) decision to have or adopt a child during fellowship?

- Yes
- No

Did you ever wish your co-fellow(s) would have waited until after fellowship to have or adopt children or would have had or adopted children prior to fellowship?

- Yes
- No

[BRANCHING LOGIC: IF YES TO ANY OF THE ABOVE 2 QUESTIONS, DISPLAY THE FOLLOWING QUESTION 🡪]

Do you feel like an alternative parental leave policy at your program would have helped with the above situation(s)?

- Yes
- No
- I do not know

[BRANCHING LOGIC: IF YES, DISPLAY THE FOLLOWING QUESTIONS 🡪]

How many children do you have?

__________________________________

(children)

What are the ages of your children? [free text]

Who is the primary childcare provider when you are at work?

- My partner
- Family member
- Friend
- Nanny
- Baby-sitter
- Day care / childcare facility
- School
- Other

What is your weekly cost for childcare on average? Your best estimate is fine.

- < $200 / week
- $200-399 / week
- $400-599 / week
- $600-799 / week
- $800-999 / week
- $1000-1200 / week
- $1200 / week
- I do not know

What is your annual household income? Your best estimate is fine.

- < $50,000
- $50,000 - 74,999
- $75,000 - 99,999
- $100,000 - $125,000
- $125,000
- I do not know

Have you or your partner had OR adopted a child during your current fellowship? If you or your partner is currently pregnant OR in the adoption process and expect to have the child during fellowship, answer "yes".

- Yes
- No

[BRANCING LOGIC: IF YES, DISPLAY THE FOLLOWING QUESTIONS 🡪]

Do you feel like you were provided with the adequate amount of information needed to plan your parental leave?

- Yes
- No
- I don't know

How many weeks of paid parental leave did you take or are planning to take? Your best estimates are fine. If you've had more than 1 child during fellowship, please use the first one to answer this question.

(example: 2 paid weeks, 3 vacation weeks, 2 elective weeks, 2 sick days, etc)

[free text]

How many unpaid weeks of parental leave did you take or are planning to take? Your best estimate is fine. If you've had more than 1 child during fellowship, please use the first one to answer this question.

__________________________________

(weeks)

How many total weeks of parental leave would you have preferred to take? If you've had more than 1 child during fellowship, please use the first one to answer this question.

__________________________________

(weeks)

Did you experience significant financial difficulties during any of your parental leaves?

- Yes
- No
- I don't know
- Not applicable as my partner or I am currently pregnant or in the adoption process.

Did you breastfeed your child? If you've had more than 1 child during fellowship, please use the first one to answer this question.

- Yes
- No
- Not applicable as my partner or I am currently pregnant or in the adoption process.

[BRANCHING LOGIC: IF YES, DISPLAY THE FOLLOWING QUESTION 🡪]

For how long did you breastfeed your child?

__________________________________

(months)

Did you ever feel discriminated against at work while pregnant?

- Yes
- No
- I don't know

Will you have to extend the length of your fellowship training because of your program's parental leave policy?

- Yes
- No
- I don't know

If you could do it over again, would you or your partner delay childbearing or adopting until after fellowship?

- Yes
- No
- I don't know

Are you or your partner planning on having a/another child during your current fellowship?

- Yes
- No
- Unsure

[BRANCHING LOGIC: IF NO OR UNSURE, DISPLAY THE FOLLOWING QUESTION 🡪]

Is your program's parental leave policy deterring you and/or your partner from having a/another child during your current fellowship?

- Yes
- No

In your opinion, when is the best time to have a child during training?

- Before medical school
- Medical school
- Residency
- Fellowship
- After training
- Unsure

Did you consider parental leave policies when ranking fellowships?

- Yes
- No

What is the amount of your current educational debt? Your best estimate is fine.

- $0
- < $50,000
- $50,001 - $100,000
- $100,001 - $150,000
- $150,001 - $200,000
- $200,001 - $250,000
- $250,001 - $300,000
- $300,001

Thank you for taking the time to complete this questionnaire. Your assistance in providing this information is very

much appreciated. If there is anything else you would like to tell us about this survey, or parental leave policy during fellowship, please do so in the space provided below.

[free text]
