# Supplementary material for: Parental leave during pediatric fellowship training: A national survey
Source: PLoS One. 2022 Dec 22;17(12):e0279447. doi: 10.1371/journal.pone.0279447 (PMC9779013; doi:10.1371/journal.pone.0279447)
Supplement: S2 File — (DOCX) [file pone.0279447.s002.docx]

**Supplement 2: Pretesting Procedures**

We performed four rounds of pretesting of the survey to reduce nonresponse and measurement error. Each round was comprised of several editing iterations. Changes were made to the survey instrument to improve clarity based on feedback and insight derived from each pretest step

.

1. Medical education leaders at the University of Colorado edited the survey for face and content validity. With their help, we tested the web survey using a variety of devices, platforms, browsers, and user-controlled settings to minimize errors faced by participants.
2. We performed cognitive interviews on six neonatology fellows at the University of Colorado as they took the survey to evaluate the survey’s cognitive and motivational characteristics.^1,2^ Specifically, we inquired about questions that were skipped, difficult to understand, or difficult to answer. Below is the script used during the cognitive interviews. It was adapted from Dillman’s “Internet, Phone, Mail, and Mixed-Mode Surveys: The Tailored Design Method”.^1^ Cognitive interviews were performed via telephone.
3. We performed retrospective interviews of junior neonatology faculty who volunteered to take the survey to evaluate the survey’s cognitive and motivational characteristics.^1,2^ After taking the survey, the faculty were asked the probing and general questions section of the cognitive interview script below either via phone or email.
4. We piloted the survey with the pediatric fellows at the University of Colorado (n=87) and obtained a response rate of 77%. With the pilot study, we ensured that each survey solicitation resulted in an appreciable increase in response rate and analyzed a free response, survey feedback question for any difficulties respondents had with the survey.

Cognitive Interview Script

Script:

Could you share your screen with me?

I’m going to email you the survey link, and I’d like you to fill it out the same way you would if it came to you at home/work, except I’d like for you to read aloud anything you would normally read to yourself. And I would like you to tell me everything you are thinking and feeling, from the moment you first see the email until you finish filling out the questionnaire and hit send. That includes your telling me anything you like or don’t like about the email and the questionnaire.

Remember to tell me what you find enjoyable and what you find frustrating from the moment you get the email until you are finished. It is important that you tell me what you are thinking and feeling while you are actually in the process of coming up with your answers. The reasons we are asking you to read and think aloud is to discover how well people work with the survey. Having people read and think aloud is a common technique in testing these surveys to assess how well they work, and to discover ways to make them better.

Now, because some people aren’t used to reading, thinking, and expressing their feelings aloud, I’d like to begin with a very short practice question. Remember to read aloud whatever it is you would normally read to yourself and to express your thought and feelings from the moment I send you the email until you are finished filling out the questionnaire. We will do this practice question via text, so I will text you this question.

- Question: How many windows are there in your home?
  - Examples of general probes:
    - What are you thinking?
    - Remember to read aloud for me.
    - Can you tell me more about that?
    - What do you mean by that?
    - Could you describe that for me?
    - Remember to tell me what you are doing.

OK, good. You’ve told me what you were thinking as you answered the question. That’s what will help us out. For the email I am about to send you, this is exactly what we would like for you to do. Remember, I want you to read aloud and tell me everything you are reading and thinking from the time I hand email you the survey until you have pressed sent on the completed questionnaire.

Are you ready?

Examples of question utilized while the survey was being completed:

- I noticed you skipped over ***, why do you think that is?
- I noticed you had to reread ***, why do you think that is?
- I noticed you looked confused when you read ***, can you tell me more about that?

Examples of questions utilized after the completion of the survey:

- Probing questions
  - Do you have any reactions to this email, either positive or negative? What are those reactions.
  - Where does it look like it is coming from and why?
  - If you received this email, would you open it? Why or why not? Does it look official? Does it look like junk mail?
  - On a scale of 1 to 5, where 1 means very easy and 5 means very difficult, how easy or difficult was it for you to figure out where to begin on the form?
  - Do you have anything else you would like to tell us that you haven’t had a chance to mention?
- General questions
  - Was it interesting?
  - Did any of the questions offend you?
  - Would you have filled out this questionnaire if it had come to you at home/work?

References

1. Dillman DA, Smyth JD, Christian LM. *Internet, Phone, Mail, and Mixed-Mode Surveys: The Tailored Design Method.* 4th ed. Hoboken, NJ: John Wiley & Sons, Inc; 2014.

2. Ruel E, Wagner III WE, Gillespie BJ. *The Practice of Survey Research Theory and Applications.* Thousand Oaks, California: SAGE Publications, Inc; 2016.
