# Supplementary material for: Parental leave during pediatric fellowship training: A national survey
Source: PLoS One. 2022 Dec 22;17(12):e0279447. doi: 10.1371/journal.pone.0279447 (PMC9779013; doi:10.1371/journal.pone.0279447)
Supplement: S3 File — (DOCX) [file pone.0279447.s003.docx]

**Supplement 3: Implementation Process and Estimating Response Rate**

Survey Implementation Process

In this study, we attempted to survey all fellows at ACGME-accredited pediatric fellowship programs in the United States. Without a centralized, national listserv of all pediatric fellows, we indirectly reached fellows through program directors (PDs) and coordinators (PCs). Prior to distributing the national survey, we sent a pre-notice to PDs and PCs informing them of the upcoming survey project and soliciting their support. Contact information was obtained off the American Medical Association’s FRIEDA website (updated at least yearly) and the ACGME program information lists. If contact information was outdated or resulted in an undeliverable prenotice, we visited the program’s fellowship website to attempt to obtain additional current contact information.

Inclusion criteria: fellows of any PGY level at the following ACGME-accredited pediatric fellowship programs in the United States during the 2019-2020 academic year (AY): Adolescent Medicine, Cardiology, Child Abuse, Critical Care Medicine, Developmental Pediatrics, Pediatric Emergency Medicine, Endocrinology, Gastroenterology, Hematology-oncology, Infectious Disease, Nephrology, Perinatal-neonatal Medicine, Pulmonology, and Rheumatology.

- Our target sample field for this manuscript is comprised of 4078 fellows

Exclusion criteria: fellows at any of the following fellowship programs

- Military-based programs as these programs may follow federal law as opposed to ACGME guidelines when it comes to parental leave (PL) policy
- Pediatric Hospital Medicine, Academic Medicine, Transplant Hepatology, and Hospice fellowships as these were not yet all ACGME-accredited during the 2020-21 AY
- Allergy and Immunology, Genetics and Metabolism, Physical Medicine and Rehabilitation, Sleep Medicine, Sports Medicine, and Neurodevelopmental Disabilities fellowships as these may have trainees whose prior training was not in pediatrics
- Child and Adolescent Psychiatry and Clinical Informatics fellowships as these are likely not governed by a department of pediatrics
- Pediatric fellowship programs at the University of Colorado as these fellows participated in our local pilot study

After the pre-notice, we sent 5 electronic survey solicitations, varied in content and approach, over a 3-week period in May and June of 2020. Emails were sent either directly to fellows for whom programs had provided email addresses or, for those that had not, to PDs and PCs to distribute to their fellows. Personalization of the contacts was used whenever possible to decrease nonresponse error. Of note, the pediatric emergency medicine (PEM) fellowship programs have a centralized PD listserv through which surveys are sent out. Our survey was approved by the national PEM survey committee, and the public survey link and survey reminders were distributed through this listserv for PDs to forward to their fellows.

On average, there was a 50% non-response rate among PDs and PCs (IQR 45.5-50.50). Additionally, 4% (IQR 2-4) of programs declined participation due to the COVID19 pandemic and 2% (IQR 0-3.5) of programs could not be contacted due to incorrect PD and/or PC contact information. For programs that did not respond, a public survey link and survey reminders were sent to their PDs and PCs asking them to forward to their fellows.

We obtained individual email addresses for 876 fellows, to whom we sent the survey directly. 46% of those fellows accessed the survey (n=407). Without email addresses for the remaining 3202 fellows in our target sample field, we were dependent on PDs and PCs to forward the public survey link. We do not know how many ever received the survey from their programs, but we do know that at least some of our respondents were from programs that did not explicitly say that they would forward the survey to their fellows. Through the public survey link, we received an additional 596 surveys. In total, 1003 fellows accessed the survey and 854 submitted fully or partially completed surveys.

Estimating Response Rate

The American Association for Public Opinion Research (AAPOR) provides the gold standard definitions for case codes and outcome rates of surveys.^1^ To determine the most conservative estimate of our response rate, which would assume that the survey reached all fellows meeting our inclusion criteria (n=4078), we can use the response rate 1 (RR1) AAPOR equation, which only denotes fully completed surveys in the numerator, and obtain a response rate of 19% (760/4078). If we use the RR2 AAPOR equation, which accounts for both partially and fully completed surveys, our response rate would be 25% (1003/4078). In contrast, in the most favorable scenario, the survey was only sent to the 1003 fellows who accessed the survey of whom 85% (851/1003) submitted partially or fully completed surveys (using the RR2 AAPOR equation) and 76% (760/1003) submitted fully completed surveys (using the RR1 AAPOR equation). In a case like ours, in which we cannot calculate a true response rate because we do not know our true denominator (number of fellows who received the survey link), some survey researchers advocate reporting a cooperation rate which excludes all noncontacts from the denominator. Using the AAPOR’s cooperation rate 1 (COOP1) equation results in a rate of 76% (760/1003), and using the AAPOR’s COOP2 equation, which includes partially completed surveys, results in a rate of 85% (851/1003). Using the cooperation rate equations corresponds to the most favorable response rate estimates. It is likely that the true response rate falls somewhere in the middle of the scenarios described.

Without a standardized listserv for graduate medical education fellows, a researcher attempting to survey the values, beliefs, or opinions of all fellows will be unable to report a true response rate. Furthermore, without a standardized listserv, a researcher would not even be able to select a randomized sample of fellows to whom to send their survey. As such, researchers may choose to limit their survey to fellows at their own institution or at a few partnering institutions. This approach would challenge the generalizability of the results given the potential variability that exists among programs (especially as it pertains to policies such as those governing PL).

Comparing Our Response Rate to the Literature

Despite all the aforementioned challenges, the fact that our survey, which did not include a monetary or non-monetary incentive, was accessed by 25% of the entire pediatric fellow population in the United States is respectable. Additionally, of those who opened the survey, 76% completed it.

According to a narrative by Johnson and Wislar in JAMA, addressing nonresponse error is more important than response rate.^2^ We systematically addressed the four sources of survey error to minimize nonresponse error:

- To eliminate sampling error, we attempted to contact all pediatric fellows in the nation.
- Supporting a lack of coverage error, our cohort’s demographics were comparable to the target population reported by the American Board of Pediatrics (ABP).^3^ However, fellows with access to the public link survey could have completed the survey more than once which would result in coverage error.
- Measurement error was minimized through careful survey design and element construction and through multiple rounds of pretesting and piloting.^4,5^
- More fellows who reported not having children skipped questions in some sections of our survey compared to fellows who reported having children. We hypothesize that fellows who have not had to use their program’s PL policy may be less familiar with the policy’s details and thus were unable to answer certain questions. On a different note, we hypothesize that many non-responders may be fellows who are not planning on having children during fellowship and thus may have thought the survey was less relevant to them. Because the ABP does not currently publish data on the percentage of fellows who have children, it is impossible to ascertain whether fellows with children were overrepresented in our cohort. We acknowledge the potential that these scenarios introduced nonresponse error into our study.

McLeod et. al. published a review of large health care provider surveys (number of respondents > 500) in the United States from 2000-2010.^6^ A total of 117 individual surveys were included. Several of their findings provide valuable context for our study. Among those studies included, there was an inconsistent approach to computation of the reported response rate. 25% used the most conservative equation (RR1) and 21% used the next most conservative equation (RR2). However, 29% excluded non-contacts from their denominator, meaning that their reported “response rate” was actually the cooperation rate, according to AAPOR definitions. Moreover, these authors report that response rates declined over the ten-year epoch, and the use of the most conservative RR1 equation was decreasing while use of cooperation rates was increasing.

Considerable attention has been given to strategies for increasing survey response rates. Two of the most common tactics are to use a mixed methods approach and to include a cash incentive.^4^ Though we initially planned to employ both strategies, we were forced to modify our approach because of COVID19. Because most academic offices around the country were closed during the pandemic and many trainees were forced to work from home when not providing direct clinical care, we believed it unlikely that an initial mailing of our survey would reach a substantial portion of our cohort. Based on McLeod’s reported association of a higher response rate with an initial outreach by mail, our dependence on email contact alone may have negatively impacted our response rate.^6^ Our decision to forego initial mail contact also precluded our inclusion of a small cash incentive with our initial contact. One study suggests that response rates to large health care provider surveys are generally <30% when incentives are not provided (McLeod et al).^6^

References

1. The American Association for Public Opinion Research. *Standard Definitions: Final Dispositions of Case Codes and Outcome Rates for Surveys.* 9th ed: AAPOR; 2016.

2. Johnson TP, Wislar JS. Response rates and nonresponse errors in surveys. *JAMA.* 2012;307(17):1805-1806.

3. The American Board of Pediatrics. Data of Subspecialty Fellows by Demographics and Program Traits. <https://www.abp.org/content/data-subspecialty-fellows-demographics-program-traits>. Published 2020. Accessed September 15, 2020.

4. Dillman DA, Smyth JD, Christian LM. *Internet, Phone, Mail, and Mixed-Mode Surveys: The Tailored Design Method.* 4th ed. Hoboken, NJ: John Wiley & Sons, Inc; 2014.

5. Ruel E, Wagner III WE, Gillespie BJ. *The Practice of Survey Research Theory and Applications.* Thousand Oaks, California: SAGE Publications, Inc; 2016.

6. McLeod CC, Klabunde CN, Willis GB, Stark D. Health care provider surveys in the United States, 2000-2010: a review. *Eval Health Prof.* 2013;36(1):106-126.
