# Supplementary material for: Parental leave during pediatric fellowship training: A national survey
Source: PLoS One. 2022 Dec 22;17(12):e0279447. doi: 10.1371/journal.pone.0279447 (PMC9779013; doi:10.1371/journal.pone.0279447)
Supplement: S4 File — (DOCX) [file pone.0279447.s004.docx]

**Supplement 4: Coding Dictionary**

**1. Impact of Laws and Culture**

- Standardization
- National policy
- National/US culture
- State law
- FMLA

**2. Impact of Policies Governing Pediatric Trainees**

ACGME, ABP, AAP, ABMS, etc.

Training grants

Governing bodies

Standardization

- Timed training VS CBME
- Confusion among fellows about the different governing bodies’ roles and jurisdictions

**3. Equity**

- Gender
- Child types (ie, natural birth, c-section, adoption, surrogacy, etc)
- Relationship types
- Childless fellows (with a lens of equity)
- Other worker types = attendings, RNs, etc
- Medical profession VS other professions
- Different policies for different fellows with unclear reasoning

**4. Burden on Co-fellows**

- Others should cover (ie, attendings, locum)
- Compensation for covering extra shifts
- Resentment, strained co-fellow relationships

**5. Burden on Parent Fellows and Their Families**

- Trainee financial burden
  - Unpaid leave, loss of salary, loans
  - Insurance (especially as it pertains to ART, freezing eggs, fertility treatment, etc.)
  - Surrogacy/adoption costs
  - Childcare (ie, realistic, on-site, help with payment, subsidized)
  - Extension of training 🡪 4^th^ year fellowship, decreased job availability
- Adverse effects on spouse relationship, spouse burden
- Adverse pregnancy/maternal/infant outcomes
  - Biological clocks
- Not enough time
  - Bonding, health (self-care, mental, physical), breastfeeding (establishment, continuation), decreased work life balance
- Extension of training

**6. Burden on Pediatric Medical Professionals**

- “We as pediatricians…”, “We in the medical field”
- Value of becoming a parent on training as a pediatrician

**7. Programmatic Approaches to Parental Leave**

- Having a PLP that exists and is written, clear, accessible, transparent, longer
- Set minimum time off
- Standardization (do not have different policies within single institution; questionable work arounds)
- Role of faculty providing coverage
- Mentoring/guidance
- Increased flexibility (to be able to accommodate unforeseen circumstances/happenings, to be able to roll over vacation or combine time amongst years of fellowship, etc)
- Institute or increase sick leave
- Supportive work environment and attendings
- Leave should really be leave and not have stipulations (ie, responsibilities, tasks, homework, etc)
- Don’t use up available paid time off
- During preg/child acquisition
  - No or decreased call shifts
  - Decreased radiation
  - Improved discretion
  - Do not overload schedule
  - Decreased discrimination
- After preg/child acquisition
  - Remote conferences
  - Lighter load upon return
  - Part time options
  - Lactation policy, pumping issues
- Expectation for academic productivity
- Staggered time off between partners
- For vs against make up time
- Differences between smaller vs larger program approaches; harder for smaller programs
- Unawareness of policy among fellows
- Confusion among all the different polices available among fellows
- Cobbling together of PL, ad hoc PL plans
- Gratefulness to program/program director for attempting to help fellow despite rules/regulations from institution/outside constraints
- Differences between fellowship and residency; different perspectives; fellowships more decentralized/defined due to being smaller but at same time more flexibility
- How the different year of fellowship are structured affect PL experience

**Coding Clarification:**

- 5vs6 = experiences leading to suggestions for improvement VS experiences due to programmatic approaches to PL
  - Chose to code as a combined 5 and 6
- 5vs4 = parent fellow burden of guilt vs co-fellow burden
  - There cannot be guilt without burden so chose to code as 4
- 3vs4 = difficult to distinguish between equity between co-fellows vs co-fellow burden
  - Try to decipher whether it has more of an equity lens or burden lens to apply primary code
- 5 implicit (2vs6) = burden of extending fellowship time implicitly due to policies governing medical trainees or institutional rules
  - As (2vs6) is implicit, code as 5
