# Supplementary material for: Parental leave during pediatric fellowship training: A national survey
Source: PLoS One. 2022 Dec 22;17(12):e0279447. doi: 10.1371/journal.pone.0279447 (PMC9779013; doi:10.1371/journal.pone.0279447)
Supplement: S5 File — (DOCX) [file pone.0279447.s005.docx]

**Supplement 5: Comparing Mean Satisfaction Scores to Aspects of How Parental Leave is Approached at Individual Fellowship Programs and to Fellow Characteristics**


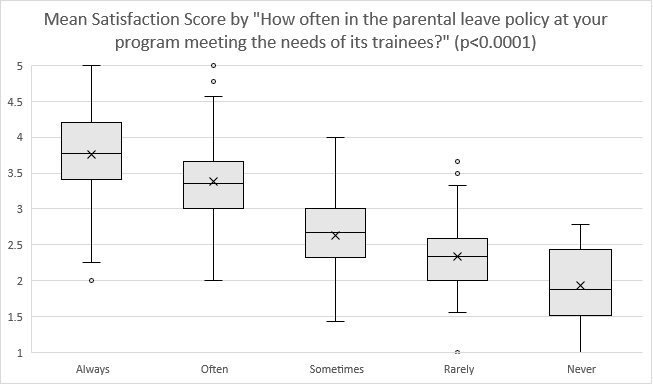


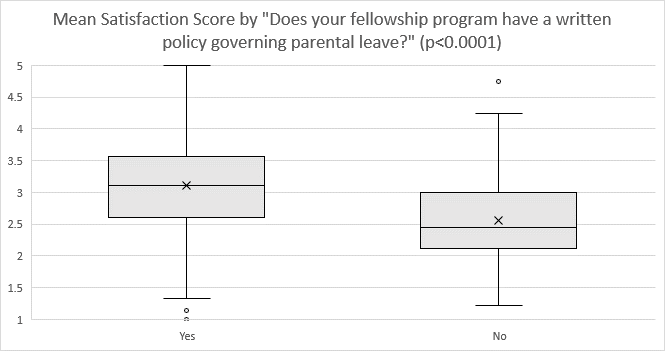


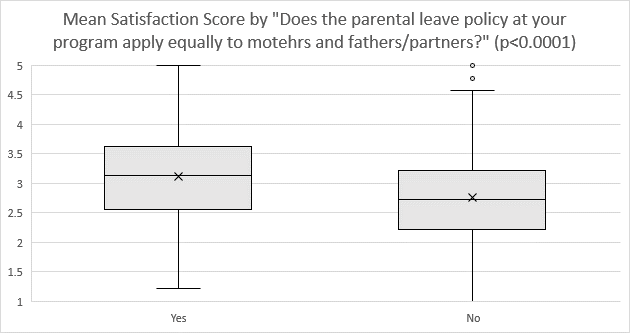


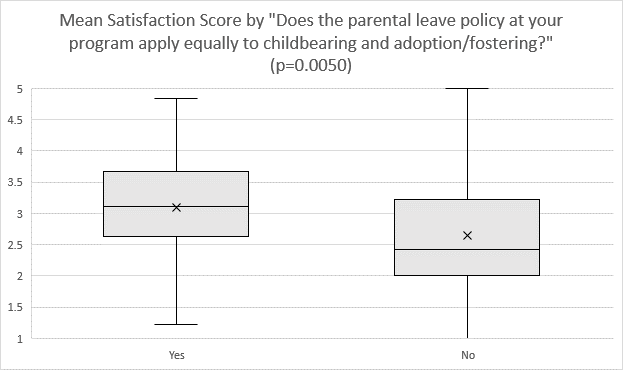


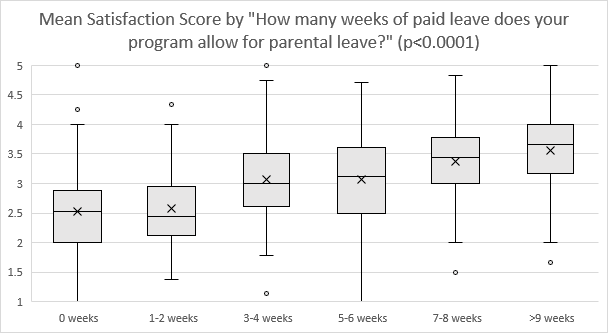


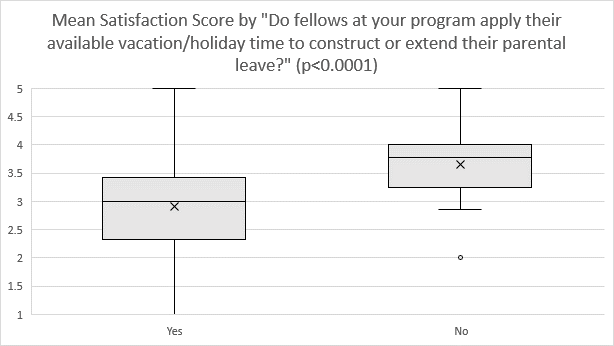


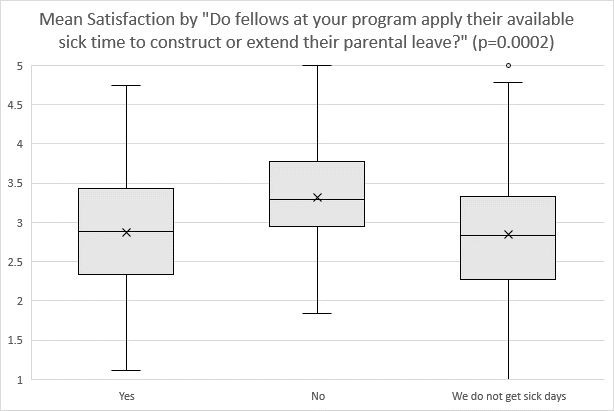


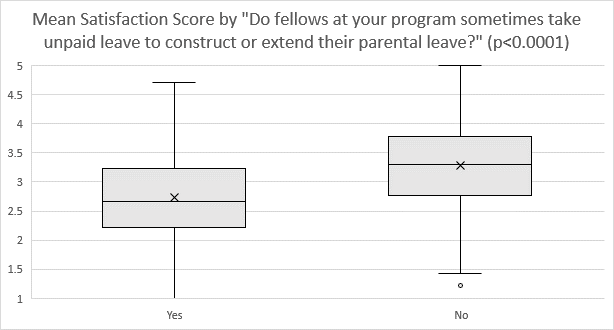


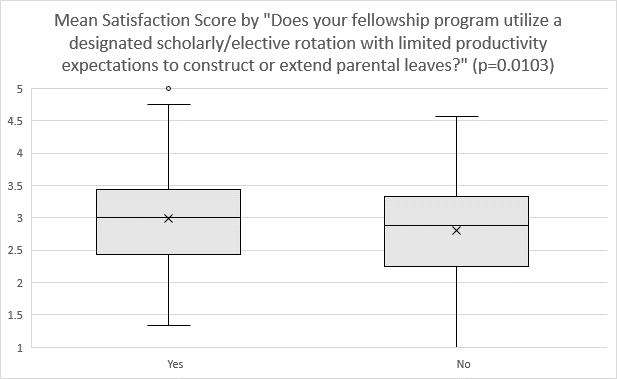


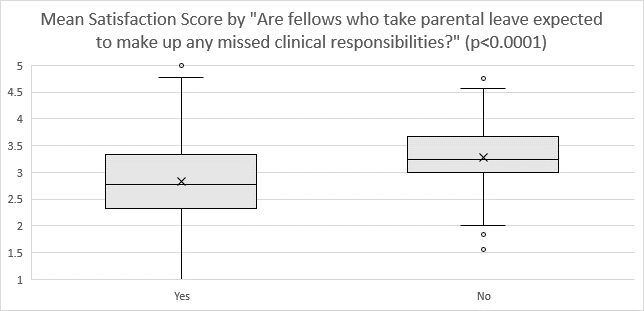


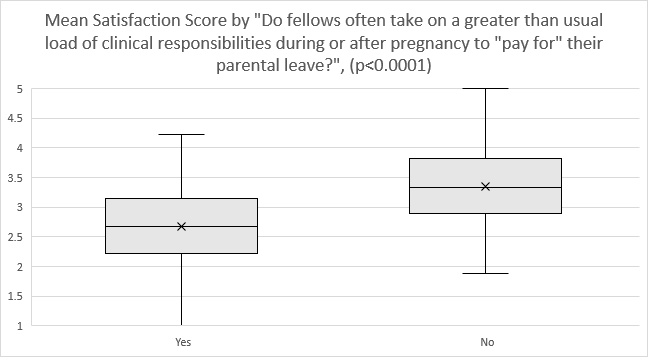


| Parental Leave Policy Satisfaction Scores by Fellow Characteristics | | |
| --- | --- | --- |
|  | Satisfaction Score^a^ |  |
|  | Mean (SD) | T-test/ANOVA p-value |
| Total (N = 626) | 3.0 (0.7) |  |
|  |  |  |
| Subspecialty |  | 0.1 |
| Adolescent Medicine | 2.8 (0.9) |  |
| Cardiology | 2.7 (0.6) |  |
| Child Abuse | 3.2 (0.8) |  |
| Critical Care | 2.8 (0.6) |  |
| Development Pediatrics | 3.1 (0.6) |  |
| Emergency Medicine | 3.1 (0.8) |  |
| Endocrinology | 2.9 (0.7) |  |
| Gastroenterology | 3.1 (0.7) |  |
| Hematology/Oncology | 3.0 (0.7) |  |
| Infectious Disease | 2.9 (0.8) |  |
| Neonatology | 2.9 (0.8) |  |
| Nephrology | 3.2 (0.8) |  |
| Pulmonology | 3.0 (0.6) |  |
| Rheumatology | 2.9 (1.0) |  |
| Gender |  | **0.05** |
| Female | 2.9 (0.7) |  |
| Male | 3.1 (0.7) |  |
| Region |  | 0.8 |
| Northeast | 3.0 (0.8) |  |
| Midwest | 3.0 (0.7) |  |
| South | 2.9 (0.7) |  |
| West | 2.9 (0.7) |  |
| Program Size |  | 0.8 |
| 1-6 Fellows | 2.9 (0.8) |  |
| > 7 Fellows | 3.0 (0.7) |  |
| Educational Debt |  | 0.3 |
| $0 | 3.0 (0.8) |  |
| <$200,000 | 3.0 (0.7) |  |
| $200,001 - $300,000 | 2.9 (0.7) |  |
| >$300,000 | 3.0 (0.7) |  |
| Have Children |  | 0.4 |
| Yes | 2.9 (0.7) |  |
| No | 3.0 (0.7) |  |
| Child during fellowship |  | **0.05** |
| Yes | 3.0 (0.7) |  |
| No | 2.8 (0.7) |  |

^a^ Satisfaction scores were calculated by summing the values of responses to each question, coded as 1 to 5 with 5 indicating higher satisfaction, then determining the mean.
